# Cardioprotective Effect of Quercetin against Ischemia/Reperfusion Injury Is Mediated Through NO System and Mitochondrial K-ATP Channels

**DOI:** 10.22074/cellj.2021.7183

**Published:** 2021-05-26

**Authors:** Ying Liu, Yi Song, Siyuan Li, Li Mo

**Affiliations:** The Center of Gerontology and Geriatrics, West China Hospital, Sichuan University, Chengdu, Sichuan, China

**Keywords:** Inflammation, Ischemia/Reperfusion, Myocardial Infarction, Nitric Oxide, Quercetin

## Abstract

**Objective:**

Quercetin (Que) is a plant-derived polyphenolic compound, that was shown to possess anti-inflammatory activity in myocardial ischemia/reperfusion (I/R) models in vivo; however, detailed mechanisms of its anti-inflammatory effects remain unclear. This study aimed to examine the effects of quercetin postconditioning (QPC) on I/R-induced inflammatory response in a rat model and evaluate the role of the mitochondrial K-ATP (mitoK_ATP_) channels and NO system in this regard.

**Materials and Methods:**

In this experimental study, hearts of male Wistar rats (250 ± 20 g) perused by Langendorff apparatus, were subjected to 30 minutes of global ischemia followed by 55 minutes reperfusion, and Que was added to the perfusion solution immediately at the onset of reperfusion. Creatine kinase (CK) levels in the coronary effluent were measured by spectrophotometry. Interleukin-1 (IL-1β), IL-6, and tumor necrosis factor-alpha (TNF-α) levels were analyzed by an enzyme-linked immunosorbent assay (ELISA) rat specific kit to assess the inflammatory condition of the myocardial tissue.

**Results:**

Our results showed that QPC significantly improved left ventricular developed pressure (LVDP) (P<0.05), and decreased the CK release into the coronary effluent vs. control group (P<0.01). The levels of IL-1β (P<0.01), TNF-α (P<0.01), and IL-6 (P<0.05) were significantly diminished in Que-treated groups when compared to the control group. Inhibiting mitoK_ATP_channels by 100 μM 5-hydroxydecanoate and blocking NO system by 100 μM L-NAME reversed the cardioprotective effects of Que.

**Conclusion:**

The findings of this study suggested that QPC exerts cardioprotective effects on myocardial I/R injury (MIRI) through inhibition of inflammatory reactions and improvement of contractility potential. Also, mitoK_ATP_ channels and NO system might be involved in this anti-inflammatory effect.

## Introduction

Acute myocardial infarction (AMI) caused as a result of coronary artery occlusion, is the most common leading cause of death and disability worldwide. At present, timely reperfusion is the major therapeutic strategy to treat myocardial ischemia; however, reperfusion itself further worsens the existent myocardial injury and may lead to extra complications such as diminished cardiac contractile function, arrhythmias, and necrosis of myocytes, a phenomenon termed "myocardial reperfusion injury" (1, 2). Therefore, in medical research, development of interventions capable of both preventing and treating ischemia/reperfusion (I/R) injuries is needed.

I/R is a complicated pathophysiological condition in which, inflammatory response, reactive oxygen-derived species (ROS) overproduction, Ca^2+^ overload, and apoptosis play central roles (3). Myocardial I/R injury (MIRI) is known to result in significant local and systemic inflammation. This inflammatory response which is triggered during ischemia, and greatly amplified during reperfusion, is characterized by increased levels of inflammatory and pro-inflammatory cytokines, including interleukin-1β (IL-1β), tumor necrosis factor-α (TNF-α), IL-6 and partially contributes to cardiac dysfunction and necrosis of cells (4-6). Increasing evidence suggests that inhibition of I/R-induced excessive inflammatory response can improve heart dysfunction caused by I/R injury (7, 8). Hence, understanding the precise mechanism of the inflammatory response is critical to improving clinical outcomes of I/R injury and designing effective therapies.

Mitochondria dysfunction is considered a major cause of cell death during I/R. Several studies demonstrated that a variety of cardioprotective strategies such as pre-and postconditioning, protects cardiomyocytes via the mitochondrial K-ATP (mitoK_ATP_) channel. Activation of mitoK_ATP_ channels maintains the mitochondrial membrane potential (ΔΨm), inhibits mitochondrial permeability transition pore (mPTP) opening, and represses mitochondrial Ca^2+^ overload, overproduction of ROS, and necrotic/apoptotic cell death (9-12). The linkage between mitoK_ATP_ channels opening and such a diverse number of the restorative processes, underscores its promising therapeutic potential in I/R injury; hence, a mitoK_ATP_ opener may improve mitochondrial function during I/R.

Nitric oxide (NO) is an important mediator in the cardiovascular system and I/R injury. Considerable evidence highlighted the beneficial roles of NO in the cardiovascular system and emphasized the link between NO and pathophysiological process of I/R injury in a way that effects of I/R injury are either mediated or antagonized by NO. Indeed, NO has both protective and detrimental role in I/R as I/R triggers a cascade involving increased NO production and leading to the excess formation of peroxynitrite (ONOO^-^) and it is accompanied by increased production of ROS, which mediates the detrimental role of NO (13, 14). The cardioprotective function of NO during I/R is due to its anti-inflammatory and antioxidant effects; furthermore, protective role of NO may be mediated through activation of the mitoK_ATP_ channels (15, 16). 

Quercetin (3,5,7,3’,4’-pentahydroxy flavone, Que) is an important member of flavonoids with the highest concentrations being found in onions and apples. A broad range of biological activities has been attributed to Que including anti-inflammatory, antioxidant, and anti-cancer activity (17, 18). Also, Que possesses the capability to reduce blood pressure and protect the heart from I/R injury (19, 20). Several studies indicated that Que postconditioning (QPC) is an effective pharmacological strategy for achieving myocardial protection against I/R injuries; however, its protective mechanism remains unclear (21, 22). 

In the present study, we investigated the cardioprotective and anti-inflammatory properties of Que and assumed that these effects were in part mediated through the NO system and mitoK_ATP _channels. 

## Materials and Methods

In this experimental study, fifty-six 12-week-old male Wistar rats weighing 250 ± 20 g, were obtained from the animal center of Sichuan University. The rats were kept in an animal room with free access to food and water, at 25˚C on a 12 hours light/dark cycle. This study conformed to the Guidelines for the Care and Use of Laboratory Animals by the National Institutes of Health (NIH Publication No. 85-23, revised in 1985), and the experimental procedures were approved by the Institutional Animal Care and Use Committee (IACUC) of Sichuan University.

### Isolated hearts and Langendorff perfusion setting 

Animals were anesthetized intraperitoneally (i.p.) with pentobarbital sodium (60 mg/kg) and heparinized (500 U/kg) to protect the heart against microthrombi. The hearts were quickly removed via thoracotomy and immersed in ice-cold Krebs-Henseleit solution (K-H). Then, the hearts were cannulated via the aorta and perfused with K-H solution that contained (in mM): 4.8 KCl, 118 NaCl, 1.0 KH_2_ PO_4_ , 1.2 MgSO_4_ , 27.2 NaHCO_3_ , 10 glucose, and 1.25 CaCl_2_ . A mixture of 95% O_2_ and 5% CO_2 _was bubbled through the perfusate, and the perfusate pH was kept in the range of 7.35-7.45. Throughout the experiment, thermostatically controlled water circulator (Satchwell Sunvic, UK) maintained the perfusate and bath temperatures at 37˚C. For measurement of interventricular pressure changes, a saline-filled latex balloon was inserted into the left ventricle (LV) and the signals were delivered to the related transducer via a connecting pressure catheter. The left ventricular-developed pressure (LVDP) was considered a cardiac contractility index.

### Induction of ischemia and reperfusion

Each experiment lasted 100 minutes in total. All groups of isolated rat hearts underwent a 15-minutes stabilization period. In all groups, after the stabilization period, the hearts were exposed to global ischemia for 30 minutes, followed by 55 minutes of reperfusion period with K-H solution at 37˚C. An immediate decline in CF at the onset of index ischemia and the recovery of the CF upon reperfusion served as evidence of effective coronary occlusion and reperfusion (23, 24). 

### Exclusion criteria

In the Langendorff apparatus, the isolated hearts were excluded from the test if their baseline coronary flow (CF) and LVDP were lower than 7.5 ml/minutes and 70 mmHg, respectively. Also, the hearts with weak contractions or with arrhythmias were excluded from the experiment and replaced with another one. The exclusion (and replacement) rate for groups was as follows: Sham=0; control=1 heart; EL-C receiving group=0; Que receiving groups=1 heart; 5-HD receiving group=2 hearts; Que plus 5-HD receiving group=1 heart; L-NAME receiving group=1 heart; and Que plus L-NAME receiving group=1 heart. The weak contraction or arrhythmias may be related to the failure in surgical procedure.

### Experimental protocol

**Fig.1:**
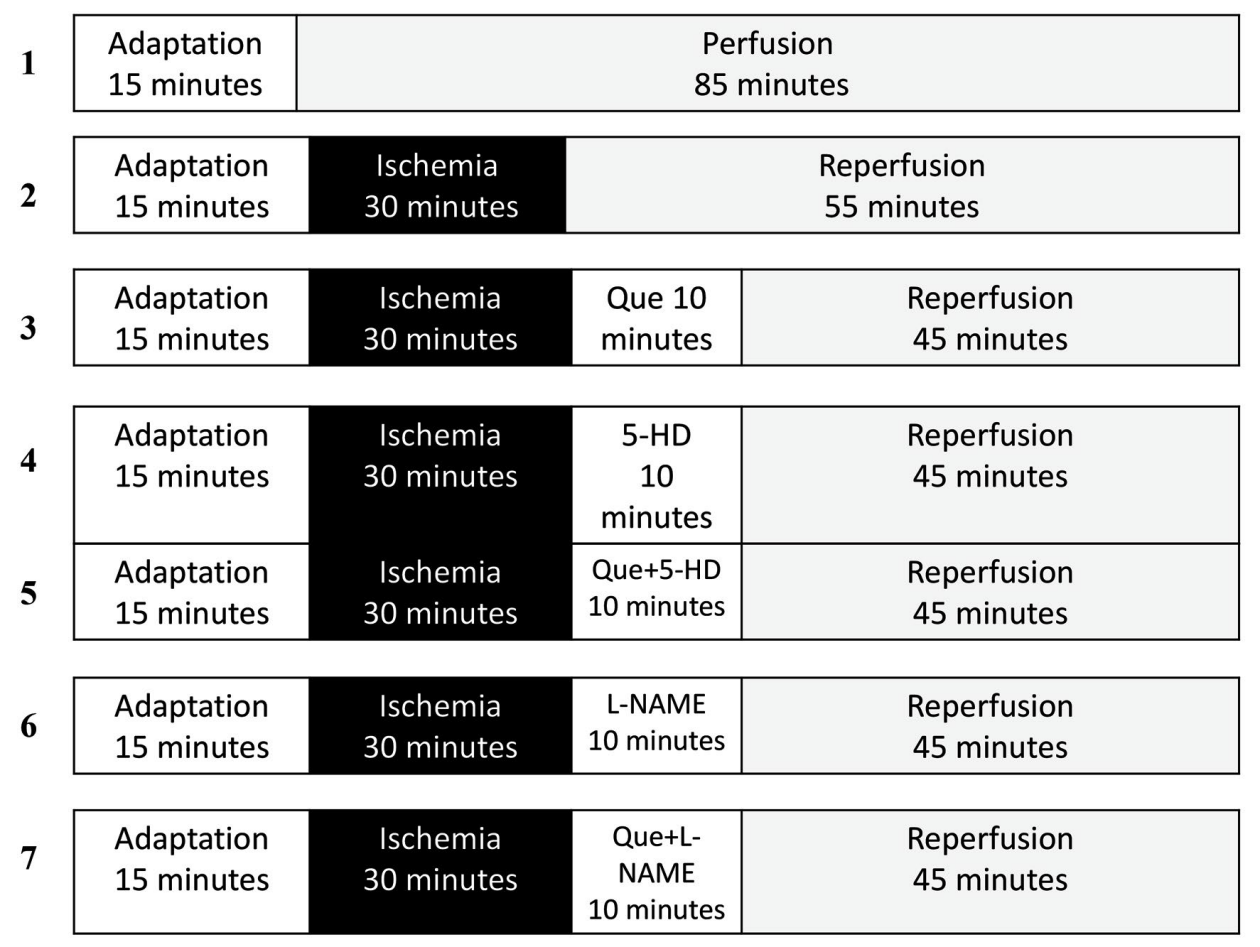
Experimental protocol. Except the Sham, which was perfused constantly for 85 minutes, the remaining hearts were subjected to 30 minutes of global ischemia followed by 55 minutes of reperfusion. 100 nM of Que was administered for 10 minutes at the onset of reperfusion. 5-HD (100 µM) or L-NAME (100 µM) was also administered for 10 minutes at the onset of reperfusion with Que absence or presence (n=7 per group). Que; Querctin, 5-HD (5-hydroxydecanoate); mitoK_ATP_ channel blocker, and L-NAME (L-nitro-arginine methyl ester); NO synthase blocker.

The fifty-six male Wistar rats were divided into eight groups (n=56, 7 per group, Fig .1):

i. Sham: in which the isolated hearts did not undergo ischemia, and were continuously perfused with a normal K-H buffer.

ii. Control: in which after the surgical preparation and 15 minutes stabilization, the isolated hearts were subjected to a 30 minutes ischemia and 55 minutes reperfusion with a normal K-H buffer.

iii. Cremophor-EL: in which the condition was similar to the control group except that the hearts were perfused with a K-H solution containing 0.1 % Cremophor-EL for 10 minutes at the onset of reperfusion.

iv. Quercetin: in which the condition was similar to control group except that the hearts were perfused with a K-H solution containing 100 nM Que for 10 minutes at the onset of reperfusion.

v. 5-HD: in which the condition was similar to control group except that the hearts were perfused with a K-H solution containing 100 μM 5-hydroxydecanoate (5-HD, as a mitoK_ATP_ channel blocker) for 10 minutes at the onset of reperfusion.

vi. Quercetin plus 5-HD: in which the condition was similar to the control group except that the hearts were perfused with a K-H solution containing both 100 μM 5-HD and then, 100 nM Que, for 10 minutes at the onset of reperfusion.

vii. L-NAME: in which the condition was similar to the control group except that the hearts were perfused with a K-H solution containing 100 μM L-NAME (as a NO synthase blocker) for 10 minutes at the onset of reperfusion.

viii. Quercetin plus L-NAME: in which the condition was similar to the control group except that the hearts were perfused with a K-H solution containing both 100 μM L-NAME and then, 100 nM Que, for 10 minutes at the onset of reperfusion.

There were no significant differences between EL-C and control groups. Due to this reason we did not consider Cremophor-EL group in the analysis of results.

### Creatine kinase release measurement

The coronary effluent was collected 10 minutes after the beginning of reperfusion, and samples were stored at -80˚C. Ischemic injury was measured based on the creatine kinase (CK-MB) activity. The CK-MB activity was determined spectrophotometrically by using commercial kits brought from Roche Diagnostic (Mannheim, Germany). The absorbance of CK-MB solution was read at 340 nm. The results were reported in Unit/l.

### Preparation of tissue homogenates

At the end of each experiments, the hearts (LVs) were separated and the ischemic zones were sampled, immediately frozen in liquid nitrogen and stored at -80˚C. Approximately 0.5 g of ventricular tissue was cut into pieces in about 5 ml of ice-cold lysis buffer containing (mM/ml): 1.0 KH_2_ PO_4_ , 1.0 KCL, 50 Tris-HCl (pH=7.4), 1.0 EDTA, 1.0 NaF, 1.0 Na_3_ VO_4_ , and 1% Triton 100X and protease inhibitor cocktail (Sigma-Aldrich, USA) and then homogenized with a Polytron PT-10/ST homogenizer. The homogenates underwent centrifugation at 10,000 g for 10 minutes at 4˚C. The obtained supernatants were removed from the homogenates quickly frozen at -80˚C. The Bradford method was used for determination of the concentration of proteins and cytokine activity in supernatants.

### ELISA for measurement of tissue levels of cytokines

The supernatant levels of IL-1β, TNF-α, and IL-6 were determined using an enzyme-linked immunosorbent assay (ELISA) rat specific kit according to the manufacturer’s protocol (Bender Medsystems, Austria). Pro-inflammatory cytokines concentrations were quantified relative to a standard curve. The optical density (OD) of each well was read at a wavelength of 450 nm. TNF-α, IL-1β, and IL-6 levels were expressed as picograms per milligram of total protein.

### Statistical analysis

Data are presented as means ± standard deviation (SD). Statistical comparisons between experimental groups were made using ANOVA with Tukey multiple comparison test. A P<0.05 was considered statistically significant.

## Results

### Quercetin postconditioning improved cardiac contractility during myocardial ischemia/reperfusion

**Fig.2:**
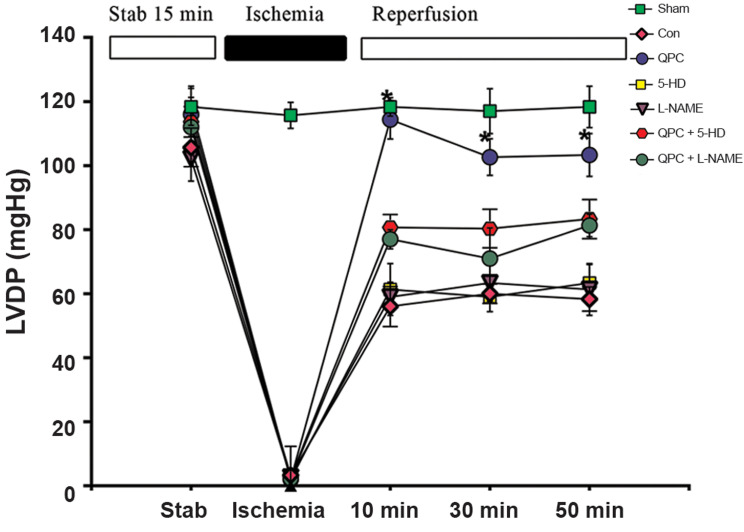
Left ventricular developed pressure (LVDP) changes in all experimental groups. The data were expressed as mean ± SD (n=7 per group). QPC; Quercetin postconditioning, *; P<0.05 QPC vs. control group, and min; Minutes.

LVDP was applied to evaluate alterations in cardiac function. Induction of global ischemia for 30 minutes resulted in a significant decrease of LVDP in the experimental groups compared with ischemic values (Fig .2). In the 10^th^ minute of reperfusion, the recovery of the cardiac function was increased after the administration of Que to the perfusion solution in the Que-treated group against rats that were not treated with Que (control or 5HD-treated, L-NAME treated, respectively). The LVDP was significantly higher in the Que-receiving group as compared with the control group (P<0.05). The recovery of myocardial function due to QPC was completely abolished by 5-HD. Similarly, administration of L-NAME eliminated the effects of QPC on LVDP.

### Quercetin postconditioning decreased the release of creatine kinase during myocardial ischemia/ reperfusion 

**Fig.3:**
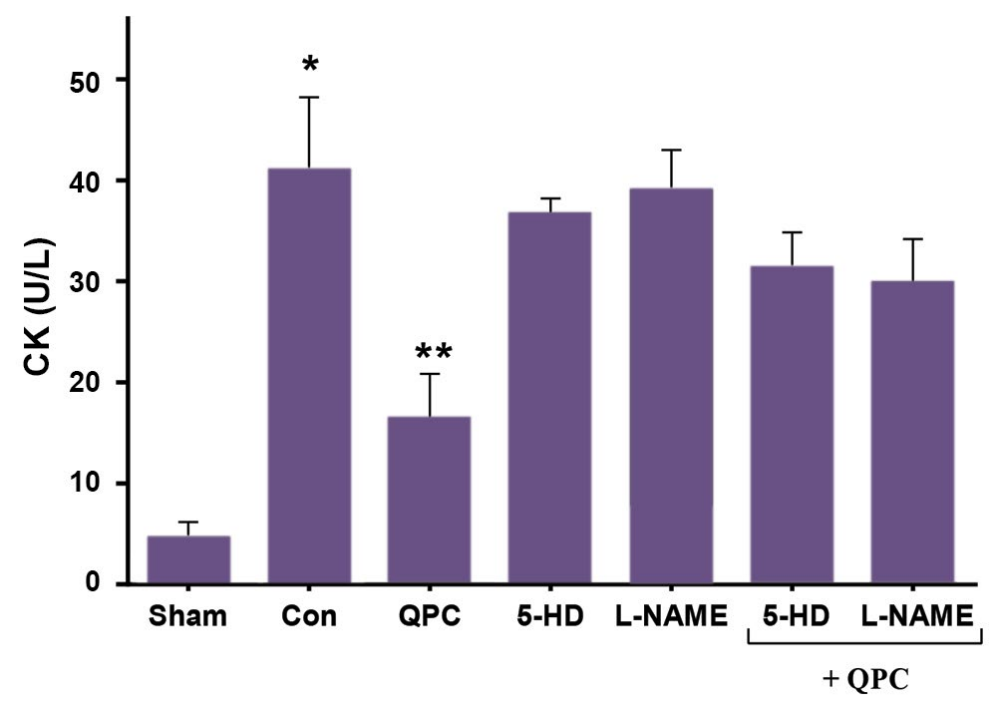
CK release during reperfusion 10 minutes in each experimental group. Data are presented as mean ± SEM (n=7 per group). *; P<0.01 vs. Sham group; **; P<0.01 vs. control, QPC; Quercitin postconditioning, 5-HD; mitoK_ATP_ channel blocker, L-NAME; NO synthase blocke, CK; Creatine kinase, and Con; Control.

The activities of CK were used to assess the injury of the myocardium. Levels of CK in the coronary effluent in I/R group were significantly increased compared with the sham group (P<0.01). The increased levels of CK were significantly attenuated by QPC (P<0.01, Fig .3). The effects of Que were abolished by the addition of the NO inhibitor, L-NAME, and the mitoK_ATP_ channel blocker, 5-HD. However, there was no significant difference in CK level between the control, and 5-HD group and L-NAME group and 5-HD+QPC group, L-NAME+QPC group.

### Quercetin postconditioning decreased tissue levels of tumor necrosis factor-alpha during myocardial ischemia/reperfusion

**Fig.4:**
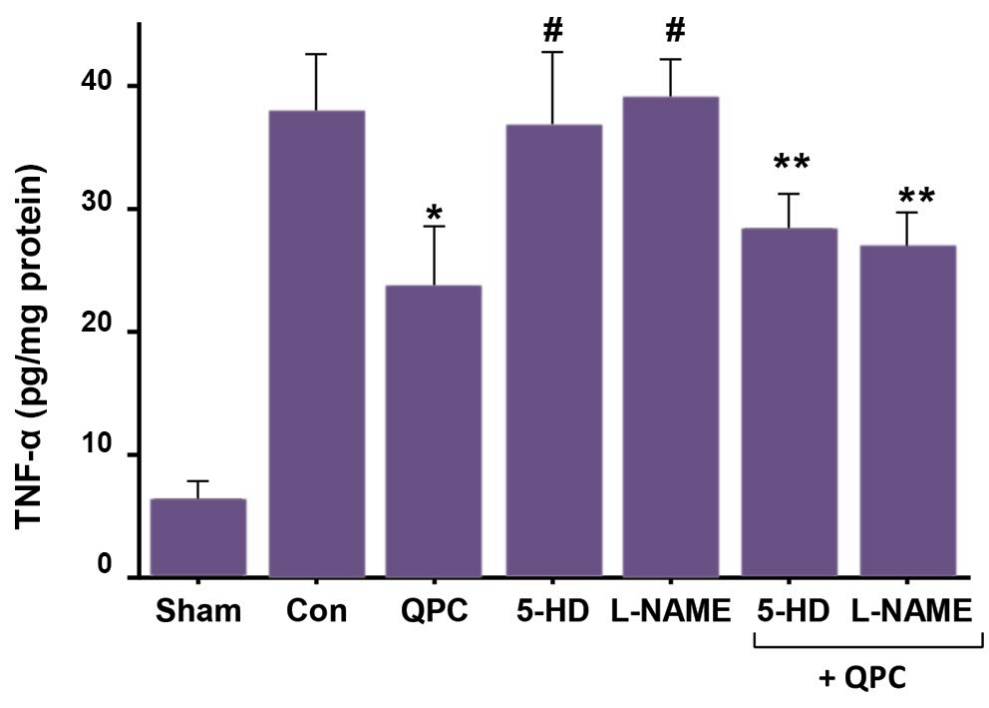
TNF-α level after reperfusion in each experimental group. Data are presented as mean ± SEM (n=7 per group). *; P<0.01 vs. control group, ^#^ ; P<0.05 vs. QPC; **; P<0.05 vs. control group. QPC; Quercitin postconditioning, 5-HD; mitoK_ATP_ channel blocker, L-NAME; NO synthase blocker, TNF-α; Tumor necrosis factor-alpha, and Con; Control.

The results are shown in Figure 4. TNF-α level in the QPC group was significantly (P<0.01) decreased compared to the control group, and similar results were seen for 5-HD+QPC, L-NAME+QPC groups compared with the control group (P<0.05). Blocking the mitoK_ATP _channels using 5-HD and blocking the mitoK_ATP_ channels using 5-HD, did not reverse the TNF-α-lowering influence of Que. 

### Quercetin postconditioning decreased tissue levels of IL-6 during myocardial ischemia/reperfusion

**Fig.5:**
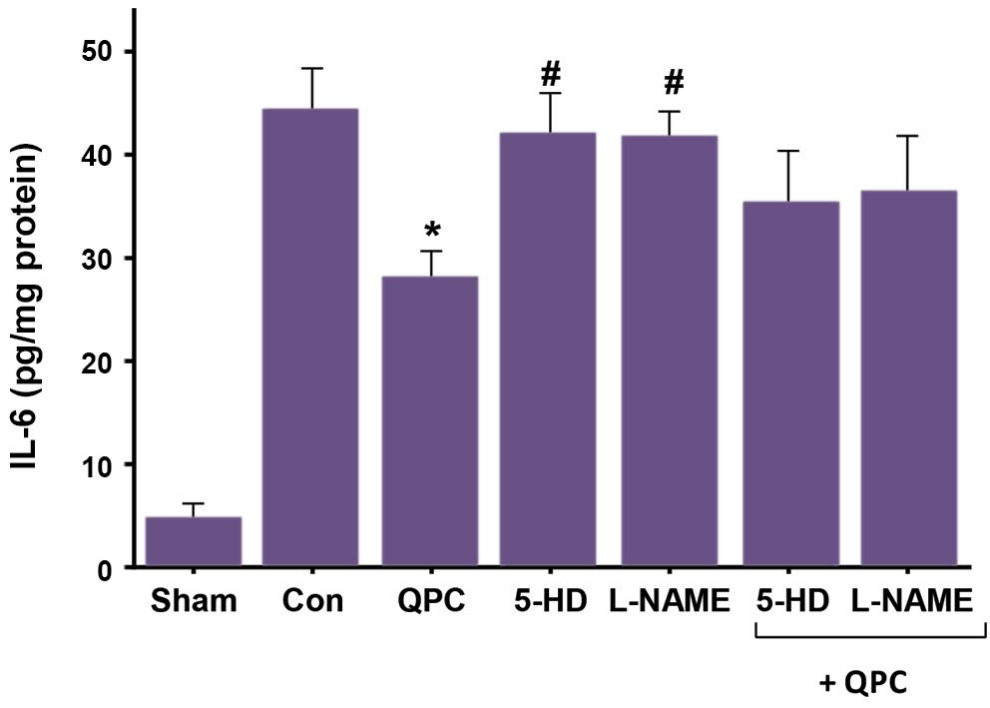
IL-6 level after reperfusion in each experimental group. Data are presented as mean ± SEM (n=7 per group).*; P<0.05 vs. control group, ^#^ ; P<0.05 vs. QPC, QPC; Quercitin postconditioning, 5-HD; mitoK_ATP_ channel blocker, L-NAME; NO synthase blocker, IL-6; Interleukin-6, and Con; Control.

QPC caused a statically significant decrease in the IL-6 level in the Que-treated group vs. untreated control hearts (P<0.05, Fig .5). No significant difference was observed between 5-HD+ QPC, L-NAME+QPC groups and untreated control hearts during reperfusion.

### Quercetin postconditioning decreased tissue levels of IL-1β during myocardial ischemia/reperfusion

**Fig.6:**
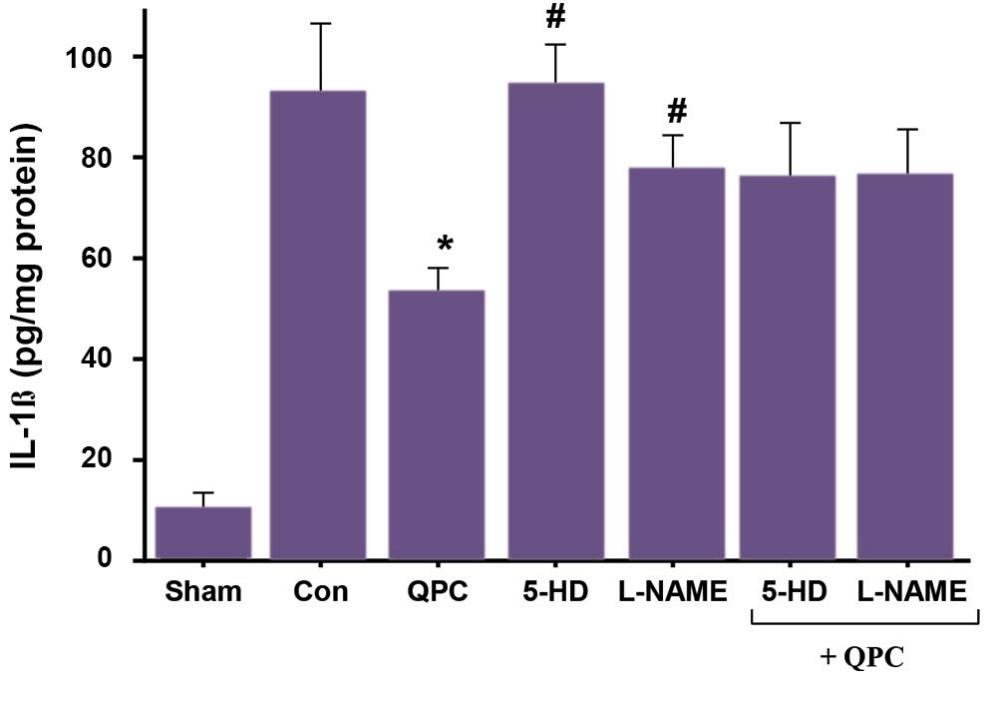
IL-1β level after reperfusion in each experimental group. Data are presented as mean ± SEM (n=7 per group). *; P<0.01 vs. control group, #; P<0.05 vs. QPC, QPC; Quercitin postconditioning, 5-HD; mitoK_ATP _channel blocker, L-NAME; NO synthase blocker, IL-1β; Interleukin-1β, and Con; Control.

QPC significantly reduced the IL-1β level in the treated group compared with control hearts that were not treated with Que (P<0.05, Fig .6). No significant difference was observed between 5-HD+QPC, L-NAME+QPC groups and untreated control hearts during reperfusion.

## Discussion

Previous studies documented a wide range of beneficial effects of Que on the cardiovascular system, such as blood pressure, left ventricular hypertrophy and myocardial I/R. In the present study, we used a rat model of MIRI to examine the protective effect of QPC against inflammatory response induced by I/R injury. We observed that QPC significantly improves decreased LVDP levels, and attenuates increased CK and pro-inflammatory cytokine levels induced by I/R injury. Importantly, the mitoK_ATP_ channels inhibitor, 5-HD and NO inhibitor, L-NAME reversed the QPC protective effect on MIRI, indicating that mitoK_ATP_ channel and NO activation could play a critical role in this regard.

In the present study, QPC significantly reduced IL-1, IL-6, and TNF-a levels compared with the untreated control group. Our results about anti-inflammatory properties of Que are consistent with a previous study done by Dong et al. (25) which indicated that Que attenuated inflammatory cytokines such as TNF-α, IL-6 and IL-1β in serum and cell supernatants in the rat heart model of I/R injury. Hong-Bo Jin showed that Que treatment inhibits inflammatory responses during MIRI through ameliorating the expression of both TNF-α and IL-10 and decreasing the levels of inflammatory cytokines in serum and cell supernatants (26). Liu et al. (19) indicated that pre-treatment with Que decreased the levels of C-reactive protein (CRP), IL-1β and TNF-α in a myocardial ischemia injury rat model. Thus, inhibitory effects of Que on the pro-inflammatory cytokines production may be a mechanism through which Que protects the heart against I/R injury.

Reperfusion of ischemic myocardium leads to an overproduction of ROS. I/R-mediated overproduction of ROS is also important for induction of inflammatory response in the infarcted myocardium (6, 27). Inflammatory reaction plays an important role in MIRI. Accumulating evidence indicated that the inflammatory response promotes the release of TNF-𝛼 from the ischemic tissue.The secreted TNF-𝛼 further stimulates the release of pro-inflammatory cytokines from infiltrating neutrophils and macrophages and produces more pro-inflammatory cytokines, such as IL-6 and chemokines (21, 28). IL-6 contributes to the extent of infarct size in the early phase of myocardial I/R (29). Also, a high levels of pro-inflammatory cytokines, such as TNF-α and IL-1β during acute myocardial ischemia, lead to exaggerated cardiac functional depression and cardiomyocyte apoptosis (30). Reducing the levels of inflammatory cytokines (IL-6, IL-1*β*, and TNF-α) could protect hearts against MIRI (31). These results demonstrate that ischemia-induced inflammatory response leads to apoptosis in cardiomyocytes, and inhibition of inflammatory response protects hearts from ischemia injury. Therefore, strategies limiting the inflammatory response has become one of the useful therapeutic adjuncts in the clinical treatment of MIRI. It is important to reduce the production of ROS by natural antioxidants such as Que because elevated levels of ROS have been the focus of considerable attention as initiators of inflammatory response. Mitochondria are widely recognized as the main source of these ROS (32). 

Several studies confirmed that conditioning strategy and pharmacological interventions mediate myocardial protection by attenuating mitochondrial dysfunction and ROS generation (9, 33). mitoK_ATP_ channels have been reported to play a key role in conditioning mediating protection (10, 34). Opening these channels modulates the synthesis of ROS and prevents Ca^2+^ overload, mitochondrial dysfunction and cell death (9, 10). In addition, it was shown that oral administration of rats with a low dose of Que exerted cardioprotective effects in isolated rat heart models during I/R injury and these protective effects of Que may be mediated through improving mitochondrial function (35). Que is known to attenuate ROS generation and protect cardiomyocytes against I/R injury (36, 37). In this study, the anti-inflammatory effects of QPC were abolished by mitoK_ATP_ channels blockade, which indicates that the anti-inflammatory effect of QPC is mediated, in part, by activation of mitoK_ATP_ channels. Consequently, since mitochondrial ROS greatly induced inflammatory responses, inhibitory effects of Que on inflammatory response may be at least partly due to attenuation of mitochondrial ROS generation (essentially, through increasing the activation of mitoK_ATP_ channels).

NO is another mediator that influences many aspects of physiological processes including inflammatory response, during MIRI (38). Although NO has been recognized as a potent biological active molecule for a variety of cardioprotective effects such as cardiac contractility and regulation of vasodilation, NO actually has both protective and detrimental role in myocardial I/R (13). In response to myocardial ischemia, enhanced amount of NO contributed to the formation of potent oxidative species peroxynitrite, the reaction product of NO with superoxide anions, which subsequently leads to significantly severe myocardial damage and mediated the detrimental role of NO (14). In contrast, NO is a reactive oxygen scavenger and by this action, increases antioxidant defenses; furthermore, NO plays a critical role in defense against MIRI through inhibition of respiratory chain. Inhibition of mitochondrial respiration by NO results in decreased oxygen-derived free radicals (16). Another mechanism that might be involved in the cardioprotective effects of NO during I/R injury is that NO increased the activity of the mitoK_ATP_ channels (39, 40). Studies suggested that the activity of the mitoK_ATP _channel was increased directly by NO. 

Independently, NO could activate GC/cGMP/PKG pathway and thereby leading to the mitoK_ATP_ channel activation (16, 40). Based on the above, NO, with its role in producing ROS, may be involved in the induction of inflammatory response during I/R injury. Our results showed that blocking NO system reversed the antiinflammatory effect of Que. The reason for the observed results could be that Que inhibits several enzymes that are involved in the generation of superoxide anions such as xanthine oxidase. Additionally, in rabbit hearts exposed to I/R injury, Que could reduce protein and mRNA expressions of NADPH oxidase 2 and inducible NO synthase (iNOS) (40). It was shown that Que modulated eNOS expression in rat isolated aorta and Que-supplemented diet modulated the expression and/ or activity of specific proteins NOX, thereby leading to increased NO bioavailability in the heart of NO-deficient rats. Also, Que scavenges superoxide anion by scavenging superoxide radicals the bioavailability of NO improves. On the other hand, Que inhibits the expression of enzymes involved in ROS generation and by this action, prevents the superoxide-mediated NO inactivation. Therefore, Que increases the bioavailability of NO, thereby it may intensify the activation of mitoK_ATP_ channels and ultimately leads to reduction of ROS generation. However, we did not detect the effect of mitoK_ATP_ channel blockade with 5-HD and NO system blockade with L-NAME on the reducing effect of Que on TNF-α. We hypnotized that there were other possible molecular mechanisms involved in this event.

## Conclusion

Our results showed that Que improved cardiac function in rats with cardiac I/R injury. The level of pro-inflammatory cytokines (TNF-α, IL-6, and IL-1) and the level of CK-MB were significantly decreased in the Que-treated group as compared with the control group. Reduction of the inflammatory response by Que is associated with its cardioprotective effects during I/R injury. The anti-inflammatory effects of Que are exerted partly through the mitoK_ATP_ channels and NO system.
